# Prenatal Alcohol Exposure and Metabolic Disorders in Pediatrics: The Role of the Oxidative Stress—A Review of the Literature

**DOI:** 10.3390/children11030269

**Published:** 2024-02-21

**Authors:** Martina Derme, Martina Briante, Mauro Ceccanti, Giuseppe Giannini, Mario Vitali, Marisa Patrizia Messina, Maria Grazia Piccioni, Alessandro Mattia, Simona Nicotera, Alba Crognale

**Affiliations:** 1 Department of Maternal, Infantile and Urological Sciences, Sapienza University of Rome, Viale del Policlinico 155, 00161 Rome, Italy; martina.derme@uniroma1.it (M.D.); mariagrazia.piccioni@uniroma1.it (M.G.P.); alba.crognale@uniroma1.it (A.C.); 2 Italian Society for the Treatment of Alcoholism and Its Complications (SITAC), 00185 Rome, Italy; 3 Department of Molecular Medicine, Sapienza University of Rome, Viale Regina Elena 291, 00161 Rome, Italy; 4 ASUR Marche, AV4, 60122 Ancona, Italy; 5 Dipartimento della Pubblica Sicurezza, Direzione Centrale di Sanità, Centro di Ricerche e Laboratorio di Tossicologia Forense, Ministero dell’Interno, 00185 Rome, Italy

**Keywords:** fetal alcohol spectrum disorder (FASD), prenatal alcohol exposure (PAE), oxidative stress, metabolic disorders

## Abstract

Prenatal alcohol exposure is responsible for increasing chronic disease risk in later life, including obesity and metabolic syndrome. Alcohol drinking may compromise endogenous antioxidant capacity, causing an increase in free radicals and reactive oxygen species in the newborn. Excessive reactive oxygen species could attack the cellular proteins, lipids, and nucleic acids, leading to cellular dysfunction. Moreover, oxidative stress could play a crucial role in the altered synthesis and release of neurotrophins and progressive mitochondrial modifications with uncontrolled apoptosis. This narrative review aims to underline the important role of alcohol abuse in oxidative stress events and consequent metabolic and neurocognitive impairments in children exposed to alcohol during gestational life.

## 1. Introduction

Prenatal alcohol exposure (PAE) is the foremost avoidable reason for congenital abnormalities and developmental disabilities and affects 2.4–4.8/1000 children [1]. PAE may also raise, in later life, chronic disease risks such as obesity, metabolic syndrome [2], and liver disease [3,4].

**Figure 1:**
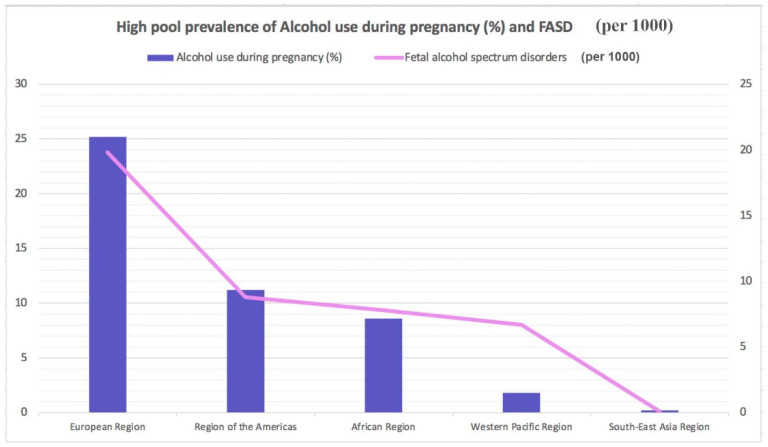
Highest pooled prevalence of alcohol use during pregnancy (%) and fetal alcohol spectrum disorders (per 1000).

Worldwide, almost 10% of pregnant women drink alcohol. The highest rate of alcoholism during pregnancy is in Europe 25.2%), followed by the American Region (11.2%), the Western Pacific Region (8.6%), the African Region (10.0%), and the South East Asia Region (1.8%). The lowest prevalence is present in the Eastern Mediterranean Region (0.2%) (Figure 1) [5]. Different Mediterranean studies measuring gestational alcohol drinking in women through the analysis of different ethanol metabolites or in the hair, meconium, or urine data showed high variability with values ranging from 3 to 4% up to more than 30% [6,7,8,9,10,11,12].

Numerous risk factors have been discovered for alcoholism in pregnancy: older age; higher socioeconomic status, salary, and educational levels; smoking; and unintended pregnancy [6,7,13,14].

Fetal alcohol spectrum disorders (FASD) is a “container” word that implies the type of circumstances resulting from PAE. FASD includes disorders such as partial fetal alcohol syndrome (pFAS), fetal alcohol syndrome (FAS), alcohol-related birth defects (ARBD), and alcohol-related neurological developmental disorders (ARND) [15,16,17,18,19,20,21,22,23,24]. Several FASD analytic guidelines have been proposed; among the most recent, Hoyme’s guidelines are quite useful [25]. These guidelines were made by a panel of expert authors who analyzed more than 10,000 children with potential FASD. They elaborated a diagnostic process that requires a multidisciplinary approach and a collaboration between pediatricians, geneticists, maternal-fetal specialists, psychiatrists, speech pathologists, physical therapists, audiologists, and ophthalmologists. The advantage of these guidelines is the possibility of elaborating a diagnosis in the prenatal period.

The global prevalence for FASD in the general population is approximately 7.7 cases per 1000 individuals. FASD prevalence is lowest in the WHO Eastern Mediterranean Region (0.1 per 1000) and highest in the WHO European Region (19.8 per 1000) (Figure 1) based on the rates of alcohol use during pregnancy. According to global epidemiological data, an estimated 1 in 13 women who drink alcohol during pregnancy will deliver a child with FASD, resulting, globally every year, in the birth of almost 630,000 children with FASD [10,11].

The PAE effects may vary depending on the frequency, quantity, pattern, duration, and timing of exposure, and it has distinct developmental consequences at different stages of organogenesis [26].

The brain is quite defenseless throughout the pregnancy [27]. The most common brain alteration is microcephaly, often associated with microencephaly [28]. Jarmasz et al. conducted a retrospective examination of 149 brains exposed to alcohol during fetal life. This study also found other important alterations: hydrocephalus, corpus callosum defects, holoprosencephaly, lissencephaly, minor subarachnoid heterotopias, and prenatal ischemic lesions [29]. Other studies revealed an overall reduction in brain volume, especially in the cerebellum, cerebrum, basal ganglia, hippocampus, caudate putamen, and thalamus [30,31,32,33,34,35]. Furthermore, studies using diffusion tensor imaging showed reduced integrity of large white matter tracts, including the hypoplastic corpus callosum, posteriorly displaced or absent. Corpus callosum alteration in FASD children is associated with changes in interhemispheric transfer of information [17,18,19,20].

Brain changes are often coexisting with craniofacial anomalies. Many FASD people display characteristic facial features such as a smooth ridge between the upper lip and nose, a thin vermillion border of the upper lip, extra crease in the outer ears, a flat nasal bridge, short palpebral fissures, smaller head size, and an upturned nose [21,22].

Most studies involve the disrupted cognitive functioning caused by alterations in brain neurodevelopment in PAE. Children with FASD show modest memory abilities, learning disabilities, language and speech delays, hyperactive behavior, impairments in the comprehension of the consequences of their actions, and inattentiveness [36]. Recent investigations refer to the theory of fetal programming and FASD as a new notion. This theory considers FASD from being a brain disease to an “entire body disorder” affecting multiple systems and organs. Indeed, PEA elevates the developing chronic conditions with potential risks, such as diabetes or cardiovascular diseases, later in life [24,25].

A robust association between congenital heart deficiencies and alcohol drinking during gestation has been shown [37,38]. Significant correlations were described with ventricular and septal/atrial defects. Children exposed to alcohol in utero may display a 1.64-fold times increased risk of being affected by subtypes of conotruncal defects such as great artery transposition [26,39,40]. Both prenatal heavy drinking and binge drinking are strongly associated with a generally increased risk of having newborns with congenital heart defects [41].

Oxidative stress seems to play a key role in the pathogenesis of both neuropsychiatric and metabolic disorders in pediatrics [42,43]. Ethanol (EtOH) can alter the endogenous antioxidant ability by depleting the levels of glutathione peroxidase and producing free radicals. Free radicals and reactive oxygen species (ROS), such as hydroxide (HO^−^) and superoxide (O^2−^) ions, are derived by O_2_ partial reduction. They can affect a cell’s structure by damaging nucleic acids, carbohydrates, proteins, and lipids. These molecules are responsible for inducing uninhibited apoptosis of fetal brain damage in children with FASD [30,44]. The FASD neuropsychiatric effects may be justified by EtOH drinking, inducing the apoptosis of serotoninergic neurons, as shown in rodent models [45].

Although many aspects participate in the pathophysiology of metabolic syndrome [46], oxidative stress due to alcohol exposure in utero plays a crucial role in the development of metabolic comorbidities such as hypertension, intolerance to glucose, and hyperlipidemia [47]. Insulin resistance could also outcome from oxidative stress, which has been shown in prenatally alcohol-exposed offspring [48].

Excessive ROS might attack the nucleic acids, lipids, and cellular proteins, leading to cellular alteration, including nonoptimal cell signaling and control of the cellular cycle, loss of energy metabolism, alteration of cellular transport mechanisms, immune activation, inflammation, and genetic mutations [49,50].

In this narrative review, our aim is to underline the major role of oxidative stress in the pathogenesis of pediatric metabolic disorders when mothers abuse alcohol during pregnancy.

## 2. Materials and Methods

A search for relevant studies has been performed in the following databases: MEDLINE, PubMed, Scopus, ScienceDirect, Google Scholar, and Web of Science. The search string has been composed by using the following keywords in various combinations: “prenatal alcohol exposure”, “oxidative stress”, “metabolic disorders”, and “fetal alcohol spectrum disorders”.

Original articles of interest, prospective and retrospective clinical studies, and review articles published in English until December 2023 have been included in this review. Relevant references cited in the included articles were also assessed for eligibility.

The investigators independently went through abstracts and titles before analyzing the full manuscripts of the retrieved papers. The clinical relevance of the papers selected after this first round of screening was assessed after a full review made by the investigators. Any discrepancies in study selection or data extraction were resolved through consensus with a third group of reviewers. This review presents evidence from the literature in a narrative format to provide a comprehensive overview of the various findings.

## 3. Results

### 3.1. Mechanism of Oxidative Stress in Metabolic Disorders

EtOH consumption in pregnancy results in an alteration of oxidative status. A recent case report [51] described increased oxidative stress in a mother abusing ethanol drinking during gestation and in her infant a few days after delivery. The FORT (free oxygen radicals test) was used, indeed, to measure the oxidative stress in the mother and her child [52]. The FORT is a colorimetric assay based on the ability of transition metals such as iron to catalyze, in the presence of hydroperoxides (ROOH), the formation of free radicals (reactions 1–2), which are then entrapped by an amine derivative, CrNH_2_. The amine reacts with free radicals, creating a colored, fairly long-lived radical cation, measurable at 505 nm (reaction 3). The color intensity correlates directly to the radical compounds and the hydroperoxide concentrations and, consequently, to the oxidative status of the sample according to the Lambert–Beer law [52]. Values superior to 330 U indicate a situation of progressing oxidative stress.

Oxidase enzymes (Nox), the mitochondria, and nicotinamide adenine dinucleotide phosphate (NADPH) are the two main apparatuses of ROS production inside the cell [53]. The Nox enzymes (Nox1, Nox2, Nox3, Nox4, Nox5, DUOX1, and DUOX2) are cell membrane proteins and Nox2-Nox3 are involved in different pathological circumstances [53]. ROS are produced in the mitochondria during oxidative phosphorylation by converting nicotinamide adenine dinucleotide (NADH) to NAD+ [54,55]. The superoxide anion and Nox2 are quickly converted by the superoxide dismutase enzyme into hydrogen peroxide (H_2_O_2_), an important signaling molecule [56,57]. Indeed, H_2_O_2_ is a potent oxidizing agent, and based on these considerations, cells are forced to secrete antioxidant peptides that convert H_2_O_2_ to water, including catalase, peroxiredoxin, thioredoxin, and glutathione (GSH) [55,58]. It is important that H_2_O_2_ production is equal to its reduction [59].

Pathological diseases such as insulin resistance, obesity, chronic inflammation, hyperglycemia, and dyslipidemia can cause overproduction of ROS [60,61]. The excessive ROS presence may elicit cellular damage, in particular, peroxidizing lipids and altering DNA [62]. Lipid peroxides, lipid peroxidation end products, may be toxic to the cell and should be removed by glutathione throughout a specific mechanism [63]. Indeed, previous investigations revealed that patients metabolically affected by the syndrome displayed greater biomarkers of oxidative damage and lower plasma antioxidant enzyme activity than healthy people [64]. Peroxidation and nitrosylation can alter nuclear acids and proteins [55]. These end products do not typically directly harm the cell [55]. However, the increase in inactive proteins may alter the cell’s capability to metabolize them, determining the activation of apoptosis and DNA damage [63]. In addition, such elevation in modified proteins reduces their function, leading to severe impairment of regular cell action [56,63]. The ROS overproduction leads to oxidative stress elevation, which also disrupts redox control and signaling, determining gene expression alteration and increasing stress response elements and growth factors by activating the apoptosis path [59,65]. Furthermore, oxidative stress may elicit profibrotic and proinflammatory pathways, which alter endothelial dysfunction and insulin metabolic signaling by promoting renal and cardiovascular fibrosis [59,66].

### 3.2. Oxidative Stress in Pediatrics after Fetal Alcohol Exposure

FASD is an umbrella expression defining all the circumstances resulting from PAE: partial fetal alcohol syndrome (pFAS), fetal alcohol syndrome (FAS), alcohol-related neurodevelopmental disorder (ARND), and alcohol-related birth defects (ARBD) [67].

The vulnerability to ethanol strongly depends on the genetic background of each individual [68], and particularly for gestation, it is not possible to establish a consumption-safe level. Indeed, the only practicable recommendation for pregnant women is to avoid alcohol use completely. Damage due to PAE can be long-lasting with no cure [69], so early management and correct identification may support prevention and alleviate the metabolic and neurological consequences affecting the FASD person later in life. FASD severity depends on the amount and drinking frequency, as well as the gestational age at which the ethanol was assumed by the pregnant woman [70,71]. Intervention services, prevention and sensibilization for the mothers could moderate the FASD incidence [72].

The fetus has inadequate or null aptitudes in alcohol metabolization and removal [73]. Indeed, the several enzymes aimed at ethanol degradation gradually elevate their actions during the various steps of gestation [52].

**Figure 2:**
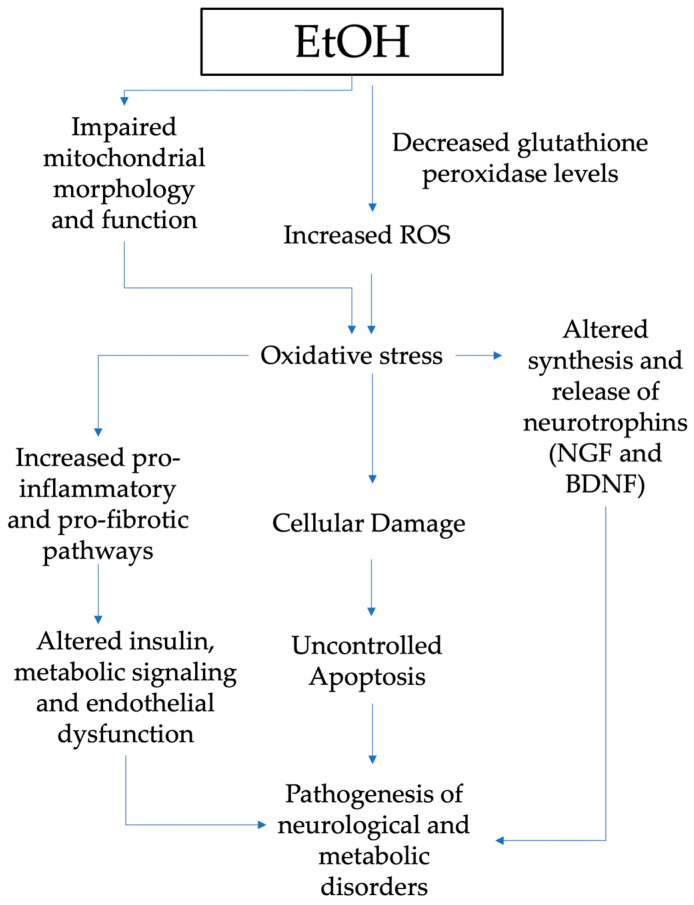
Pathogenesis of neurological and metabolic diseases and role of oxidative stress in patients exposed to alcohol in the prenatal period (EtOH, ethanol; ROS, reactive oxygen species; NGF, nerve growth factor; BDNF, brain-derived neurotrophic factor.

Figure 2 summarizes the mechanisms through which oxidative stress has a major role in the pathogenesis of neurological and metabolic diseases of patients exposed to alcohol in the prenatal period.

EtOH can alter the endogenous antioxidant ability by reducing GSH and generating free radicals, which are considered to be responsible for uncontrolled apoptosis [74,75]. Chronic and acute alcohol drinking during prenatal development also impacts mitochondrial function and morphology, another crucial cell oxidative stress source [76]. Depleted mitochondrial activity is discovered in the early postnatal stage in liver and brain tissues, including the cerebellar brain cells of prenatally exposed rats [76].

Oxidative stress has a key role in the altered synthesis and release of growth factors such as the nerve growth factor—NGF and brain-derived neurotrophic factor—BDNF [77]. Animal model studies disclosed many findings on gestational alcohol exposure’s effects on neurotrophins [78,79]. Indeed, maternal alcohol exposure during gestation affects the neurotrophins’ brain signaling pathways, as well as in target tissues for ethanol intoxication. NGF and BDNF are peptides that not only play a pivotal role in the development, survival, and function of the central and peripheral nervous systems but also regulate the pathogenesis of other problems induced by alcohol exposure [78,80,81].

The supplementation with natural compounds with antioxidant properties, such as the polyphenols extracted from vegetables, might, of course, counteract the toxic prooxidant effect of alcohol abuse during pregnancy [82,83,84,85,86,87]. Furthermore, a healthy diet during pregnancy containing proper amounts of fresh vegetables containing polyphenols, as evidenced by the Mediterranean diet, might reduce the oxidative stress induced by gestational alcohol drinking [88,89,90,91,92,93,94,95].

### 3.3. FASD and Metabolic Disorders

PAE and subsequent FASD can cause lifelong alterations in affected offspring, including a permanent imbalance in metabolic homeostasis. These effects of alcohol consumption during pregnancy could be linked to an increased risk of intrauterine growth restriction (IUGR) [96] due to blood flow impairment [97] and abnormal placentation process [98], with following catch-up growth [99]. This phenomenon is strongly correlated with the development of some features of metabolic syndrome, such as central obesity, glucose intolerance, and dyslipidemia [100].

Several studies conducted both in humans and in animal models try to underline the effects of PAE on metabolism. In 2020, Weeks et al. [101] demonstrated, with a retrospective cross-sectional study in adults with any form of FASD diagnosis, that alcohol exposure in utero increases the incidence of hypertriglyceridemia, type 2 diabetes mellitus, and lower HDL cholesterol, independently of BMI in the case of the male cohort. Female patients had, instead, an increased risk of being overweight and obese. They confirmed these data with zebrafish, a particularly suitable model considering its flexibility and anatomical similarities to humans, the presence of many evolutionary conserved pathways, and the simplicity of alcohol administration in an aqueous environment [102].

In Week’s zebrafish model, PAE had a positive correlation with elevated body mass index, increased visceral adiposity, and fasting hyperglycemia due to mild reduction in activity level, atypical organ development, and a response to diet challenge. He and colleagues [103] investigated, in a rat model, the role of PAE in the susceptibility of high-fat diet (HFD)-induced metabolic syndrome and the correlation with sex. Their study suggests that a high-fat diet seems to worsen PAE-associated neuroendocrine metabolic programming, especially in females, with a reduction in serum adrenocorticotropic hormone and corticosterone levels and enhancement in triglyceride and total cholesterol concentration, serum glucose, insulin, and insulin resistant index.

The relationship between PAE and HFD was also analyzed by Shen and colleagues [104] in 2014 when they evaluated the susceptibility of female adult offspring to HFD-induced nonalcoholic fatty liver disease (NAFLD), which is considered a liver indicator of metabolic syndrome [105]. In the group exposed to PAE and HFD, they found a decrease in serum corticosterone and an increase in serum IGF-1, glucose, and triglyceride with notable catch-up growth, higher metabolic status, and NAFLD formation. The authors suggest a “two-programming” hypothesis for the augmented risk of NAFLD in the case of prenatal alcohol exposure, in which the “first programming” consists of the intrauterine programming of liver glucose and lipid metabolic function and the “second programming” is led by postnatal adaptive catch-up growth triggered by intrauterine programming of glucocorticoid-IGF1 axis.

Yao et al. showed that EtOH drinking for one week only during pregnancy induces long-lasting harmful effects as cellular stress in adult rat offspring in association with elevated class II histone deacetylase (HDAC) proteins and SIRT2 and altered glucose regulation (increased gluconeogenesis and glucose intolerance) [106]. Dembele et al. showed that EtOH stimulates oxidative injury in the hypothalamus and decreases proopiomelanocortin (POMC) levels, which could reduce melanocortin signaling, leading to previously documented changes in body weight, food intake, and insulin sensitivity in rodents in utero exposed to EtOH [107].

In 2018, Gårdebjer and colleagues [108] investigated, in a mouse model, if periconceptional alcohol consumption, alone or in combination with a postnatal high-fat diet, determines liver dysfunction and obesity. They found out that in male offspring, PAE and a high-fat diet increase the risk of obesity and that PAE alone is correlated with microvesicular steatosis and an increase in plasma triglycerides, HDL, and cholesterol. In females, the fat mass augmented only in correlations with HFD and PAE determines an increase in LDL, cholesterol, and leptin. Using a rodent model, Al-Yasari et al. [109] showed that preconceptional alcohol exposure significantly affected the pancreatic function of the offspring, in particular insulin production and secretion and inflammation cytokine production. This results in the development of significant hyperglycemia and hypoinsulinemia, which the authors suggest could be linked to proopiomelanocortin (POMC) neuronal reductions in the hypothalamus. This abnormality alters the physiological process of POMC-regulated suppression of sympathetic neuronal systems responsible for the inhibition of pancreatic beta-cell insulin production and release and activation of parasympathetic neuronal systems implicated in the insulin release. In their study, offspring behaviors were also affected by PAE, leading to the development of increased stress and anxiety linked with epigenetic changes in several stress-regulatory genes, including POMC. Fuglestad and colleagues, in 2014, investigated the relationship between PAE and obesity. They showed that patients affected by partial FAS had the highest prevalence of being overweight (40%), while patients with FAS had the lowest prevalence of being overweight, with only 14% overweight or obese and at least one in six being underweight. It is interesting to note that even if the prevalence of overweight and or obesity was higher for both adolescent males and females with FASD compared to controls, this rate was particularly high for adolescent females (50% of the obese female patients vs. 14% of male patients) [110].

Werts and colleagues [111], in 2013, linked PAE with female overweight and lack of satiety. They suggest that alterations in brain maturation could influence the neuroendocrine signals that control reward and appetite in regions such as the hypothalamus and ventral tegmental area. This work also underlines that PAE causes inadequate micronutrient intake (low vitamin D status) and constipation, a symptom not always experienced, that could be linked to functional or structural alteration of enteric nerve, which derives from the neural crest, a target of EtOH’s neurotoxicity [112]. Another work from Amos-Kroohs et al. [113] underlines that, compared with healthy controls, children with FASD had significantly delayed acquisition of self-feeding behavior and solid food introduction. Impaired satiety and constant snacking were common and independent of medication use. The mean body mass index was significantly reduced for males but not females with FASD.

### 3.4. FASD and Cardiovascular Disease

Congenital heart defects (CHDs) are the most common congenital anomaly, with a worldwide prevalence of 9.1 in 1000 live births [114,115]. The etiology of CHDs is still unknown; most of them are due to genetic anomalies and aneuploidies. The Maternal Heart Association established that prenatal exposure to therapeutic drugs and substances of abuse, like alcohol and cigarettes, are important risk factors [116]. PAE has also been shown to be related to the occurrence of CHDs. Alcohol has severe effects on the cardiovascular system, leading to various disease states such as arrhythmias [117] and dilated cardiomyopathy [118].

The cardiotoxicity of alcohol does not interest only adult consumers. According to the Centers for Disease Control and Prevention (CDCP), about 10% of pregnant women mentioned drinking fluently, and approximately 50% of them mentioned binge drinking, which increases the risk of FASD [119]. The proportion of children with CHDs among children with FASD is almost 67% [120]. The molecular mechanisms can explain the illness, but the American teratogenic effects of PAE are still poorly understood because of the complexity of alcohol effects and the correlation with timing, amount, and duration of exposure, as well as genetic susceptibility [121].

Zhong and colleagues showed that different levels of alcohol exposure in utero have different effects on histone protein acetylation and subsequent expression of some genes related to heart development (i.e., GATA4, Mef2c, and Tbx5). Low levels of alcohol increased histone protein H3 acetylation but did not significantly impact heart development. In contrast, high levels of alcohol-induced both H3 acetylation and important gene expression changes. These findings suggest that alterations to histone modifications are a potential mechanism for alcohol-related CHDs [122]. Genetic and epigenetic factors affect in utero development of the fetus and can lead to abnormal phenotypic manifestations. It has been scientifically proven that increased oxidative stress caused by substance and alcohol abuse, smoking, nutritional imbalances, and other diseases like obesity and diabetes during pregnancy may induce placental dysfunction, metabolic alterations, and consequent onset of traditional cardiovascular risk factors [123].

In animal models, EtOH exposure during the development of fetal anatomical structures leads to oxidative stress, apoptosis, mitochondrial dysfunction, and activation of the proinflammatory pathway and results in structural heart defects, cardiac hypertrophy, fibrosis, apoptosis, oxidative stress, cardiac channelopathies, and contractile dysfunction [124,125]. Ercan et al. showed a close correlation between higher total oxidant status (TOS), total antioxidant status (TAS), and oxidative stress index (OSI) and cyanotic CHD, whereas no significant correlation was found between the oxidative status and non-cyanotic CHDs and control group [126]. In fact, it is interesting to note that many studies suggest that CHDs more closely related to the PAE are conotruncal defect subtypes such as d-transposition of the great arteries and tetralogy of Fallot [41,127,128,129]. PAE is related not only to CHD but also to cardiac rhythm alterations in the absence of structural anomalies or cardiac channelopathies. Onesimo and colleagues reported two interesting cases of children affected by FASD according to Hoyme’s criteria [25]; the first case showed uniform and isolated premature ventricular contractions (PVCs), and the second case showed frequent premature atrial contractions (PACs) and short runs of ectopic atrial tachycardia [39]. Therefore, screening for arrhythmias in children affected by FASD without structural CHDs must be executed. Understanding the molecular mechanisms underlying PAE-induced cardiotoxicity in human cells can help guide the development of management and therapeutic strategies for children affected by FASD and cardiac disease. Hwang et al. investigated the effects of alcohol on mitochondrial features and transcriptomic and metabolomic profiles in cardiomyocytes derived from human induced pluripotent stem cells (hiPSC-CMs) [130].

By modeling chronic alcohol exposure-induced cardiotoxicity in hiPSC-CMs, they showed that EtOH causes decreased mitochondrial membrane potential and mitochondrial content, decreased mitochondrial function, and altered expression of related genes [130]. EtOH also modified the glycolytic process and carbohydrate metabolic process as well as a reply to hypoxia, increased glycolysis, decreased mitochondrial function, and increased oxidative stress [130]. Therefore, an upregulation of T-cell chemotaxis has been shown as a potential causal link to proinflammatory response [130]. Further studies are needed to better understand the mechanisms of alcohol cardiotoxicity and teratogenicity in order to prevent the dramatic effect of PAE on the offspring of mothers consuming alcohol.

## 4. Conclusions

Exposure to ethanol in utero elevates oxidative stress biomarkers, determining damage to DNA, proteins, lipids, and alterations of endogenous antioxidants.

Although many aspects participate in the pathophysiology of metabolic syndrome, oxidative stress due to alcohol exposure in utero plays a crucial role in the development of metabolic comorbidities such as high blood pressure, increased glucose intolerance, insulin resistance, and hyperlipidemia.

This review underlines the important impact of alcohol on oxidative stress processes and consequent metabolic and neurocognitive impairments in kids and adolescents affected by FASD. Further, it is necessary to initiate investigations on a larger scale to elucidate other physiopathological mechanisms inducing neuropsychiatric and metabolic disorders in pediatrics when exposed to alcohol in utero.
